# The Different Facets of Extracellular Calcium Sensors: Old and New Concepts in Calcium-Sensing Receptor Signalling and Pharmacology

**DOI:** 10.3390/ijms19040999

**Published:** 2018-03-27

**Authors:** Andrea Gerbino, Matilde Colella

**Affiliations:** Department of Biosciences, Biotechnology and Biopharmaceutics, University of Bari, 70121 Bari, Italy

**Keywords:** Ca^2+^-signalling, microelectrode, fluorophore, exocytosis, cAMP signalling, parathyroid extracellular-Ca^2+^-sensing receptor (CaR), cancer, apoptosis, ischemia/reperfusion, hypertrophy, heart, cardiomyocytes

## Abstract

The current interest of the scientific community for research in the field of calcium sensing in general and on the calcium-sensing Receptor (CaR) in particular is demonstrated by the still increasing number of papers published on this topic. The extracellular calcium-sensing receptor is the best-known G-protein-coupled receptor (GPCR) able to sense external Ca^2+^ changes. Widely recognized as a fundamental player in systemic Ca^2+^ homeostasis, the CaR is ubiquitously expressed in the human body where it activates multiple signalling pathways. In this review, old and new notions regarding the mechanisms by which extracellular Ca^2+^ microdomains are created and the tools available to measure them are analyzed. After a survey of the main signalling pathways triggered by the CaR, a special attention is reserved for the emerging concepts regarding CaR function in the heart, CaR trafficking and pharmacology. Finally, an overview on other Ca^2+^ sensors is provided.

## 1. Introduction

Over the years, the intracellular Ca^2+^ signal has emerged as the most intensely studied second messenger pathway [1]. Ca^2+^ is the most versatile intracellular messenger, not only in the cytoplasm but also in diverse subcellular compartments, such as mitochondria [2], nuclei [3], lysosomes [4], and the endoplasmic reticulum [5]. The cloning of the extracellular Calcium-sensing receptor (CaR) 25 years ago by Brown et al. [6] paved the way for a heightened understanding of the mechanisms used by Ca^2+^ to signal in the microenvironments outside the cell. Thus, Ca^2+^ acts as a multipurpose messenger, exerting effects at the cellular, subcellular, and extracellular levels. A very interesting and comprehensive list of reviews on the CaR has been recently published by leading experts in the field of extracellular Calcium-sensing and signalling [7]. Other key topics, such as the structure-function relationships [8] and role of the CaR in cancer [9], have also been very recently covered elsewhere and will be named here in the corresponding paragraphs. The readers are thus referred to these fine reviews for a complete understanding of the CaR’s function in cell physiology and pathology [10].

The present review will examine the potential conditions under which extracellular Ca^2+^ concentration ([Ca^2+^]) may change in the local microenvironment outside the cell, with a particular emphasis on the experimental approaches utilized to measure ex vivo and in vitro such Ca^2+^ fluctuations. These extracellular Ca^2+^ changes are sensed by the CaR (and other Ca^2+^ sensors) and transduced by means of different signalling pathways that modulate organ-specific physiological processes. Over the last few years, new concepts have emerged regarding the CaR’s role in cancer and cardiac physiology and pathology. Also, exciting news have come into view in the fields of CaR signalling, trafficking, and pharmacology. In addition, it is now clear that a number of different Ca^2+^ sensors share important tasks with the CaR. Therefore, the aim of this review is to summarize for the reader of *International Journal of Molecular Science* these new and exciting notions.

## 2. Extracellular Ca^2+^ Microdomains

Serum-ionized Ca^2+^ concentration (~1.4 mM) is usually precisely regulated by a number of cooperative organs, including parathyroid glands, bones, kidney, and intestine [11]. However, this tight modulation does not prevent local changes in extracellular Ca^2+^, as those have been identified in the microscopic volume of interstitial fluid surrounding cells of many tissues [12]. This scenario has become increasingly important because the amplitude and the shape of these extracellular Ca^2+^ fluctuations represent an autocrine/paracrine cell-to-cell communication form based on the activity of extracellular Ca^2+^ sensors.

Many factors have been proposed in the onset of such significant extracellular Ca^2+^ changes. At first, we will evaluate how the activity of the cells, in terms of intracellular Ca^2+^ signalling events, can be rapidly translated in asymmetrical Ca^2+^ changes. Then, we will analyze how the exocytosis of Ca^2+^-loaded vesicles and the synchronous opening of voltage-gated Ca^2+^ conductances perturb Ca^2+^ levels in the close proximity of a cell.

When closely packed to form a functional tissue, cells delimit small diffusion spaces. Within this context, cells themselves represent major sources and/or sinks for Ca^2+^ during intracellular Ca^2+^ signalling events as a result of the activation of Ca^2+^ efflux (e.g., by the plasma membrane Ca^2+^-ATPase and/or Na+/Ca^2+^ exchanger) and Ca^2+^ influx (e.g., by store-operated channels, SOCs) pathways at the plasma membrane, respectively. Differential dynamics or a polarized distribution of these Ca^2+^ influx/efflux mechanisms [13,14,15,16] are required for the onset of significant extracellular Ca^2+^ microdomains. Our group showed that stimulation with carbachol, a Ca^2+^-mediated agonist, resulted in asymmetrical fluctuations in local extracellular Ca^2+^ levels in a polarized epithelium.

**Figure 1:**
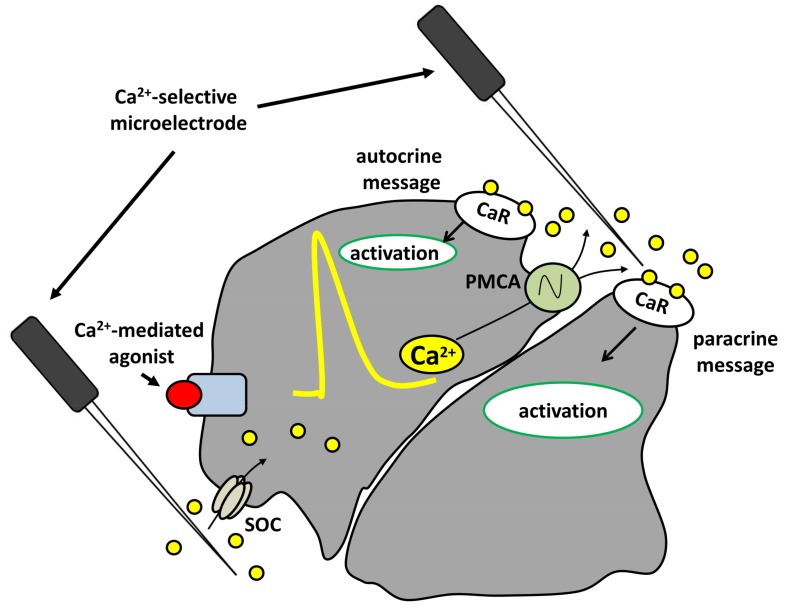
Schematic representation of intercellular communication mediated by the calcium-sensing Receptor (CaR) in polarized epithelial cells. When a Ca^2+^-mediated agonist (red circle) binds a specific receptor, Ca^2+^ concentration (yellow line) increases within the cytosol. Extrusion of Ca^2+^ via the plasma membrane Ca^2+^-ATPase (PMCA), localized at the apical membrane, might activate the CaR expressed on cells in close proximity (paracrine message) or on the same cell (autocrine message). The asymmetrical changes in extracellular Ca^2+^ are the result of the polarized localization of PMCA and store-operated channels (SOCs) at the apical and basolateral membranes, respectively. These fluctuations can be recorded with Ca^2+^-sensitive microelectrodes. Modified from [29].

Using Ca^2+^-selective microelectrodes, we measured a substantial plasma membrane Ca^2+^-ATPase (PMCA)-dependent local increase in the extracellular Ca^2+^ concentration at the apical face and a similar store-operated channel (SOC)-dependent depletion at the basolateral side of amphibian acid-secreting cells (Figure 1). Thus, in intact epithelia, asymmetrical changes in extracellular Ca^2+^ reflect the polarized distribution of Ca^2+^-handling mechanisms in the plasma membrane [17]. Of note, PMCA is highly expressed at the apical membrane of these cells where it shares a strategic localization with the extracellular CaR [17]. In fact, these carbachol-induced changes in extracellular Ca^2+^ act as paracrine/autocrine messages sufficient and essential to strictly regulate tissue-specific functions (e.g., alkaline secretion, pepsinogen secretion, and water flux) independently of intracellular [Ca^2+^] changes [18,19].

Secretory granules are characterized by very high intraluminal Ca^2+^ concentrations [20,21,22]. Among others, insulin granules of rat insulinoma contain ionized Ca^2+^ in a range between 60 and 120 mM [23]. Thus, it does not come as a surprise that exocytotic events can significantly change extracellular Ca^2+^ in the microenvironment immediately surrounding the cells. Recently, we showed using Ca^2+^-microelectrodes that stimulation of insulin secretion by high glucose and other secretagogues (glibenclamide, forskolin/IBMX, and ATP) induced a delayed elevation of extracellular Ca^2+^ within rat insulinoma (INS-1E) β-cell pseudoislets [24]. The exocytotic extrusion of Ca^2+^ through Ca^2+^-loaded vesicles released in the extracellular environment has also been demonstrated in different cell types, such as salivary gland cells [25], bovine adrenal medullary cells [26], neurohypophyseal nerve endings [27], and sea urchin eggs [28].

In addition to the above-mentioned mechanisms, excitable cells express a different kind of voltage-gated Ca^2+^ conductances that, during their physiological activity, might influence extracellular Ca^2+^ levels [30,31,32]. It has been demonstrated that, in the central nervous system, the synchronized opening of voltage-gated Ca^2+^ channels (VGCCs) elicited significant reductions in extracellular Ca^2+^ levels [33,34]. We and others have shown that in mouse islets of Langerhans and INS-1E pseudoislets, glucose stimulation depleted the concentration of Ca^2+^ in the extracellular milieu (of about 500 μM) as a consequence of Ca^2+^ influx through a VGCC at the plasma membrane [24,35,36]. Transient reductions were also recorded in rat neocortex [33] and in the cardiac muscle [37].

## 3. Measuring Fluctuations in Extracellular Ca^2+^ Levels

Real-time measurement of extracellular Ca^2+^ changes in the proximity of the plasma membrane (e.g., intercellular spaces) is a technically challenging experimental maneuver. In fact, researchers must deal on the one side with the lack of proper tools to directly access these restricted compartments, and on the other side with the quantification of small Ca^2+^ changes (few hundreds micromolar) over a high extracellular Ca^2+^ background (physiologically around 1.1–1.4 mM). Over the years, many different experimental approaches to measure extracellular Ca^2+^ fluctuations have been proposed in a number of different tissue preparations, although each one of them has its own drawbacks in terms of either sensitivity or spatial resolution.

Microelectrode-based electrophysiological approaches have been applied to diverse cell and tissue types to monitor extracellular Ca^2+^ changes. Early studies directly measured changes in extracellular Ca^2+^ concentration in the rat cerebellum using double-barreled Ca^2+^-selective micropipettes. During repetitive stimulation of the central nervous system, extracellular Ca^2+^ was significantly reduced, indicating that Ca^2+^ modulation in brain microenvironments may be a significant parameter in the behavior of neuronal ensembles [38].

The vibrating probe technique uses a low-resistance Ca^2+^-sensitive electrode vibrating in proximity of the surface of cells or tissues in order to measure small extracellular Ca^2+^ gradients (for more technical details refer to [39,40]). Self-referencing, low-resistance vibrating Ca^2+^ microelectrodes were shown to non-invasively quantify, in real time, local extracellular ion gradients with high sensitivity and square micron spatial resolution. Ca^2+^-selective self-referencing electrodes have been used extensively to show Ca^2+^ flux at different regions of hair cells, cells of the zona pellucida, and the central nervous system [41,42,43,44].

As described in the previous section, we extensively used Ca^2+^-selective microelectrodes to directly record the profile of agonist-induced changes in extracellular [Ca^2+^] in the restricted domain of both the intact amphibian gastric mucosa [17] and in the microenvironment immediately surrounding INS-1E cells organized as pancreatic pseudoislets [24].

Finally, Mupanomunda and colleagues used in situ microdialysis techniques in anesthetized Wistar rats to monitor extracellular [Ca^2+^] in the sub-epithelial spaces of the intestine [45] and kidney [46]. In these studies, an increased load of [Ca^2+^] in the gut lumen or perturbations in whole-animal Ca^2+^ homeostasis were shown to cause significant changes in the external [Ca^2+^] underlying the epithelium of these tissues.

Ca^2+^-sensitive dyes have shown extensive utility in the study of the spatio-temporal dynamics of Ca^2+^ fluctuations. These tools, compared with electrophysiological techniques, are more sensitive and provide for a better time resolution and more reliable access to limited spaces. However, these approaches are performed in free extracellular Ca^2+^ (thus, non-physiological conditions) in order to avoid saturation of the fluorophores.

Ca^2+^-sensitive metallochromic indicators (antipyrylazo III and tetramethylmurexide) were used to estimate extracellular Ca^2+^ dynamics in intact beating hearts. These indicators revealed significant depletion of external Ca^2+^ following the synchronous opening of a Ca^2+^ influx pathway during contraction [37,47,48]. Later on, Tepikin and Petersen introduced the droplet technique [49,50,51], an elegant approach to measure extracellular Ca^2+^ changes elicited by the activity of the PMCA. This method employs Fluo-3 under Ca^2+^-free media conditions to quantify extracellular Ca^2+^ changes in small clusters of exocrine gland cells contained in a small droplet of solution (20–50 times the volume of the cells). Extracellular Ca^2+^ was also measured in cell clusters digested from exocrine glands (pancreas, submandibular gland) and placed in a small experimental chamber containing Ca^2+^ green-1 bound to high molecular weight dextran beads in Ca^2+^-free solution [25,52].

Ca^2+^-sensitive probes conjugated to a hydrophobic tail have been created to cross the external leaflet of the cell membrane and directly measure extracellular Ca^2+^ levels. A classic example is Ca^2+^-Green-C-18, the fluorophore conjugated with a lipid derivative that has been successfully used to record Ca^2+^ extrusion and fluctuations ^2+^inside the T-tubules [53]. In smooth muscle cells, the Ca^2+^-sensitive indicator Fura-C18 was used to measure extracellular Ca^2+^ changes near the plasma membrane. The same dye was used to compare the extracellular Ca^2+^ changes recorded with signals measured from the bulk cytosol [54,55]. In addition, Fura-C18 has been used to directly measure near-membrane extracellular [Ca^2+^] changes in HEK-293 cells, showing that Ca^2+^ extruded to the extracellular space by PMCA, as a consequence of intracellular Ca^2+^ events, activates a positive feedback through the CaR [56].

## 4. The Extracellular Calcium-Sensing Receptor (CaR)

### 4.1. Discovery and Cloning of the Extracellular Calcium-Sensing Receptor

The first hints about the existence of an extracellular Ca^2+^ sensor come from a very early study (1966) correlating serum Ca^2+^ concentration and parathyroid hormone (PTH) secretion in whole animals [57,58]. About 10 years later, further evidences corroborated such a correlation in isolated glands and dispersed parathyroid cells [11,59,60,61,62,63,64].

The direct link between extracellular [Ca^2+^] changes, PTH secretion, and intracellular Ca^2+^ signals was finally demonstrated by Nemeth and Scarpa [65], while the actual molecular identification of the Ca^2+^ sensor had to wait until 1993, when Brown and colleagues cloned a peculiar G protein coupled receptor (GPCR) from the bovine parathyroid glands [6] and named it “extracellular Calcium-sensing receptor”. This receptor, here named CaR, is also referred to as CaSR, after its gene name, or more recently CaS, as recommended by NC-IUPHAR [66].

### 4.2. Structural Features of the CaR

The extracellular calcium-sensing receptor belongs to class C (or 3) of the GPCR superfamily, which consists of eight metabotropic glutamate receptors (mGlu1-8), two type B metabotropic-aminobutyric acid receptors (GABA_B1_ and GABA_B2_), three taste receptors (T1R1-3), a promiscuous l-α-amino acid (GPRC6A) receptor [67], and seven orphan receptors [68]. The primary structure of human CaR consists of four main parts: a large N-terminal extracellular domain (ECD) (612 amino acids) [69], a cysteine-rich domain, a seven-transmembrane domain (TMD, 250 amino acids), and a 216-residue carboxyterminal (C)-tail [70].

The ECD contains a large bilobed, nutrient-binding Venus Flytrap (VFT) domain related to bacterial periplasmic binding proteins which has been crystallized in several members of GPCR class C (mGlu1 [71], mGlu3 and mGlu7 [72], and the GABA_B2_ subunit [73]). It seems that upon Ca^2+^ (or other agonists) binding, the open cleft of the VFT closes inducing, in turn, conformational changes in both the TMD and the intracellular domains that are believed to initiate signal transduction [74,75].

The functional, highly glycosilated CaR proteins reside on the cell membrane as homodimers [76,77] or hetero-dimers with other class C receptors (mGluR [78] or GABA_B_ [79]). While non-covalent interactions across the dimer interface [80] are essential for dimer formation, the intermolecular disulfide between two conserved cysteines of the VFTs (C129 and C131 [81,82,83]) seems not to influence the formation of dimer but to stabilize it [84]. Critical for CaR expression and activity seem to be other three intra-molecular disulfides formed by cysteines in the ECD of the CaR, including C60–C101, C358–C395, and C437–C449 [85]. More recently, Brauner-Osborn showed that one allosteric site per CaR dimer is sufficient for achieving the modulatory effect of positive allosteric modulators, while prevention of activation in both 7TM domains is needed in order to obtain complete inhibition by negative allosteric modulators [86].

The nine-cysteine structure domain (found in all members of the GPCR family C but the GABA B receptor) serves as a linker between the ECD and TMD and is essential for the CaR signalling capacity as demonstrated by the consequences of its removal [87]. The intracellular domain of the CaR shows a low degree of conservation among species [88] except for residues located at the beginning of the C-tail (863–925) that are crucial for surface expression [89] and those (960–984) involved in the interaction with downstream proteins [8,90,91]. As for other GPCRs, CaR activity can be regulated via phosphorylation by different protein kinases, such as PKC [76], which plays a significant role in the negative regulation of the CaR’s activity and the generation of CaR-induced intracellular Ca^2+^ oscillations. On the other hand, PKA [92] is involved in the intracellular retention of the receptor [93]. For detailed reviews on the structure of the CaR and new insights on ligand binding pockets, see [8,70,94]. The structural requirements for processes such as bias and allosteric modulation of the CaR have been analyzed by recent works [95]. In addition, a number of papers have highlighted the importance of the potential Ca^2+^ binding sites and their relevance for associated diseases [8,96,97,98,99,100,101,102]. Recently, the first high-resolution crystal structure of the ECD of the human CaR bound with Mg^2+^ has been proposed [102]. In this work, the authors found a high-affinity tryptophan derivative in the crystal structure of the CaR, which seems to play a role in potentiating the function of the receptor. The proposed CaR co-activation operated by extracellular Ca^2+^ and amino acids might have a wide impact for the regulation of the receptor in districts of our body that are constantly exposed to both CaR agonists, such as the intestine. An exhaustive review about the recent insights regarding the structure and functional cooperativity of the CaR (and other members of cGPCRs) in health and disease has been published by the group of Jenny Yang [103].

### 4.3. CaR Promiscuity: Many Orthosteric Agonists and Allosteric Modulators Concur in Modulating CaR Function at Multiple Sites

Although, as the name states, the main ligands of the CaR are extracellular Ca^2+^ ions, which bind to the receptor with a high positive cooperativity, the CaR is also able to respond to other extracellular stimuli (see Table 1), such as inorganic di- and tri-valent cations, including Sr^2+^, Ba^2+^, Gd^3+^, Al^3+^ [11,104], various organic polycations, such as the polyamines spermine and spermidine [105], and basic polypeptides, including polylysine, polyarginine [11,106], and amyloid β-peptides [107]. The CaR has also been shown to be activated by some aminoglycoside antibiotics, such as neomycin and gentamycin [108,109]. These ligands, referred to as type I calcimimetics or orthosteric agonists, can stimulate the receptor also in the absence of Ca^2+^ or any other ligands.

The other class of CaR agonists is represented by type II calcimimetics (or allosteric modulators), composed by ligands which bind to a site different from that of the orthosteric agonists, thus altering the receptor conformation and, as a consequence, the affinity and/or the signaling capacity of the orthosteric agonist. This action can be exerted in a positive (calcimimetics) or a negative direction (calcilytics).

Belonging to this class of CaR agonists are natural l-amino acids, especially aromatic [111,112,113] and glutathione analogs [114,115], which synergize with extracellular Ca^2+^. The receptor’s activity is also negatively modulated by low extracellular pH [116] and high ionic strength [117].

A detailed review on the binding sites of orthosteric and allosteric agonists of the CaR has been recently published by Zhang and colleagues [8].

Among the allosteric modulators of the CaR, there are drugs with a high therapeutical potential: first, second, and third generation calcimimetics [118,119,120,121].

After the first generation of calcimimetics, which is represented by phenylalkylamine derivatives, such as NPS R-568 and NPS R-467 [122], the second generation reached the clinic: cinacalcet is the only CaR modulator currently used in clinical practice to reduce parathyroid glands activity in patients under dialysis for end-stage renal disease [123,124,125]. The third generation calcimimetics, which comprise the dibenzylamine calcimimetic, *R*,*R*-calcimimetic B, and the structurally distinct calcimimetic AC-265347, appear to have enhanced tissue-selective effects on PTH and calcitonin release in rats [126,127] most probably via biased allosteric modulation of the CaR’s activity [128] (see below).

Allosteric antagonists or calcilitics, such as NPS-2143 and Calhex 231, antagonize the stimulatory effects of extracellular Ca^2+^ and are currently in clinical trials for treating osteoporosis [129].

In addition to affecting the signaling capacity of the CaR, allosteric CaR modulators have been shown more recently to act as pharmacochaperones that rescue cell surface expression of mutant CaRs that are retained intracellularly (see the paragraphs below).

### 4.4. Trimeric G Proteins and Signalling Pathways Activated by the CaR

**Figure 2:**
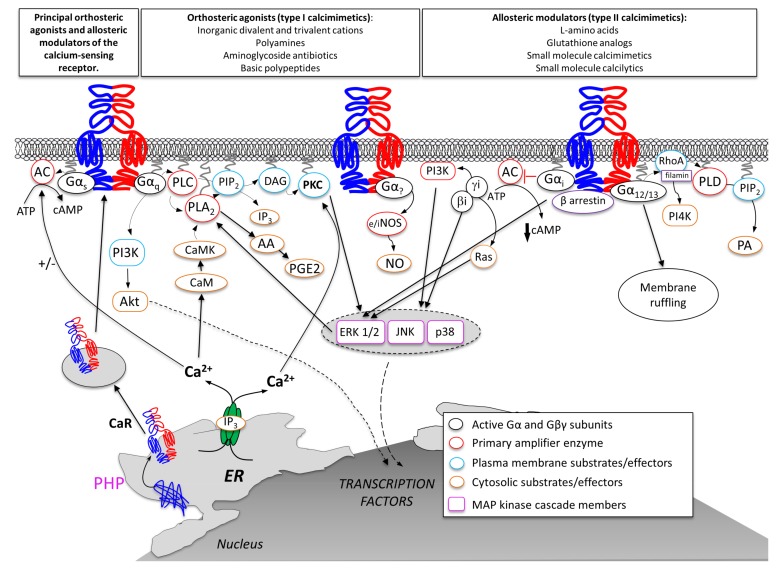
Schematic illustration of intracellular transduction cascades activated by orthosteric agonists or allosteric modulators on the CaR. Modified from [110].

Besides its ability to sense changes in multiple physiological parameters via interaction with different ligands, the CaR has also been shown to activate a wide array of different intracellular pathways (Figure 2) (for comprehensive reviews see [130,131]).

#### 4.4.1. Gq/G11

Most of the studies available in the literature on CaR physiopathological functions in diverse tissues of the body have demonstrated that this receptor, as shown in parathyroids [61,132], mainly interacts with the Gq/11 heterotrimeric G protein, inducing in turn the activation of phospholipase C (PLC)/inositol-1,4,5-trisphosphate (InsP3)/Ca^2+^ mobilization and activation of PKC and mitogen-activated protein kinase (MAPK) pathways [133]. These intracellular events cause a decrease in parathyroid hormone (PTH) secretion and reduction in renal tubular Ca^2+^ reabsorption [133].

PKC has been described to phosphorylate specific CaR residues and attenuate signalling and/or activate the mitogen-activated protein kinase (MAPK) cascade (ERK1/2, p38 MAPK, JNK), ultimately causing phosphorylation and activation of ERK1/2 [134]. CaR-induced and Gq-mediated activation of phospholipase A2 and phospholipase D have also been reported [135]. Also, activation of the Akt pathway by Gq (via phosphatidylinositol 3-kinase, PI3K) has been described as a determinant in CaR-mediated cell migration [136] and metalloproteases production [137]. The activation of the intracellular Ca^2+^ signalling pathway is also responsible for a Gi/o-independent inhibition of cyclic AMP synthesis mediated by Ca^2+^-induced inhibition of adenylyl cyclase isoforms 5, 6, and/or 9 [138,139,140]. For a recent review see also [141].

#### 4.4.2. Gi/o

Early studies have led to the suggestion that the CaR can interact with the pertussis-toxin-sensitive inhibitory G protein, Gαi/o, causing inhibitory control of PTH secretion [142,143] although the matter is still debated [144]. More recent work has clearly demonstrated CaR-mediated inhibition of adenylyl cyclase (AC) by a pertussis toxin (PTX)-sensitive mechanism. In gastric mucosa, CaR activation led to PTX-dependent alkaline and pepsinogen secretion [18]. In CaR-expressing HEK-293 cells, CaR stimulation quickly reversed or significantly prevented the agonist-induced rise in cytosolic cAMP via a dual mechanism involving pertussis-toxin-sensitive Gi and the CaR-stimulated increase in intracellular Ca^2+^ level [139]. The beta gamma subunits of the Gi/o protein have also been described as mediators of CaR -induced ERK1/2 phosphorylation via Ras and MAPK activation [134]. More recently, the role of beta-arrestin in Gi/o protein-dependent activation of (ERK) and c-Jun N-terminal kinases (JNK) has been directly demonstrated [145]. Gi activation by the CaR has also been reported to induce activation of phosphatidylinositol 4-kinase (PI4K) [146,147].

#### 4.4.3. G12/13

G12/13 pathways have been described in different cell types to differentially modulate CaR-mediated intracellular Ca^2+^ kinetics.

Both early and recent studies have described the capacity of aromatic amino acids to induce intracellular Ca^2+^ oscillations via a G12/13 /Rho/ filamin-A pathway, which in turn induce Ca^2+^ influx from the extracellular fluid via the TRPC1 channel opening [148,149,150]. On the contrary, in pre-osteoblasts, a G12-dependent dephosphorylation of CaR residue T888 by phosphatase PP2A [151,152] seems to be involved in the appearance of the sustained Ca^2+^ increases necessary to activate chemotaxis [153]. In bone, CaR-mediated G12/13 activation positively modulates osteoblast differentiation [154] and negatively modulates osteoclastogenesis [155]. Importantly, CaR-dependent G12/13 signalling seems to be also implicated in the spreading of breast and prostate cancer cells [156].

#### 4.4.4. Gs

In some cell contexts, such as human breast cancer [157] and pituitary cells [158], the CaR has been shown to modulate hormone secretion by activating Gs. Interestingly, a CaR-induced Gs-independent mechanism for the increase of cyclic AMP synthesis has been recently identified in human fetal lung [159].

Finally, a role for nitric oxide (NO) in mediating CaR action on ion channels has been recently demonstrated in the vasculature [160,161,162,163,164].

### 4.5. Physiopathological Significance of CaR: Old and New Aspects

As the name suggests, the main physiological function of the CaR is to sense subtle changes in serum Ca^2+^ and maintain systemic Ca^2+^ homeostasis within a narrow range via the oppositely directed changes in secretion of PTH, which in turn modulate the equilibrium between Ca^2+^ uptake and conservation via a direct action on kidney, bone, and thyroid C cells [165] and indirect actions on intestine. Also, the CaR controls calcitonin secretion from thyroidal C-cells upon detection of variations in [Ca^2+^]ext (for a review, see [11]).

When the extracellular Ca^2+^ concentration is reduced, CaR-induced parathyroid hormone (PTH) secretion is elicited by the parathyroid glands. Consequences of PTH release are: (a) reduced excretion of Ca^2+^ by the kidney; (b) increased intestinal absorption of Ca^2+^, which is mediated by PTH-induced synthesis of the active vitamin D metabolite 1,25-dihydroxyvitamin D; and (c) net release of skeletal Ca^2+^ under sustained PTH secretion. All these tasks cooperate to increase serum Ca^2+^ back to physiological levels. On the other hand, when serum Ca^2+^ levels are increased over the physiological range, the receptor is activated inhibiting PTH synthesis and secretion [166].

The physiological significance of the CaR in tissues involved in systemic Ca^2+^ homeostasis is well-highlighted by the identification of mutations of the CaR gene that cause several inherited disorders of extracellular calcium sensing (for a review, see [167]).

Familial hypocalciuric hypercalcemia (FHH) and neonatal severe hyperparathyroidism (NSHPT) [168] are caused by loss-of-function mutations of the CaR gene, whereas autosomal dominant hypocalcemia (ADH) and Bartter Syndrome type V [169] occur from gain-of-function mutations of the CaR [170,171,172].

Besides its expression in tissues involved in Ca^2+^ homeostasis, such as thyroid glands, kidneys, and bone, the CaR has been identified in an astonishing number of diverse cell types (reviewed in [11] and [173]), i.e., brain [174], pancreas [175], stomach [89], liver [176], and heart [177,178]. In these tissues, the CaR has been shown to regulate a variety of cellular processes, such as secretion, differentiation, and gene expression. For recent reviews on this topic see also [74,179].

#### 4.5.1. CaR Involvement in Cancer

The massive impact of the CaR in such a wide array of cellular processes, including proliferation, differentiation, migration, adhesion, and apoptosis foresees a role for this receptor in many aspects (incidence, progression, recurrence, and lethality) of various cancers (e.g., parathyroid, pancreatic, prostate, breast, colorectal, ovarian, gastric, skin, and neuroblastoma) [9,180]. However, mutations of the CaR are not early events in tumorigenesis. Still, an increasing amount of literature is concordant on CaR participation in various aspects of the neoplastic progression. In this line, a number of papers evaluating the role of CaR polymorphisms in the risk, incidence, recurrence, or lethality of different kinds of cancer has been published [9].

The CaR is associated with both anti- and pro-proliferative signals. Thus, it can either prevent or promote tumorigenesis depending on the cellular context and the type of cancer [180]. Tissue-specific alterations of CaR expression level, functional activity, or coupling to signaling pathways might explain such opposing behavior.

In tissues such as parathyroid or colon, CaR inhibits cell proliferation and induces terminal differentiation and apoptosis. In tumors of these organs (and more recently also of the gastric mucosa), the observed and progressive reduction in CaR expression (e.g., from normal tissue to carcinomas) grants malignant potential, indicating for the receptor and the physiological significance of its activation a putative role as tumor suppressor [181,182,183,184,185]. Accordingly, when taking also CaR functional activity into consideration, inactivating mutations of the CaR or calcilytics might induce hyperplasia, while calcimimetics have the opposite outcome [186,187]. This scenario is extremely important assuming that the CaR is a novel target for the prevention/treatment of these tumors. However, as reported throughout this review, the incredible number of CaR interactors (both extracellular and intracellular) increases the complexity associated with this kind of pharmacological approach.

Indeed, some of the first evidence connecting extracellular Ca^2+^ and tumors was the inverse correlation between dietary Ca^2+^ level and risk of colorectal cancer [188]. This effect seemed to be mediated by the CaR and potentiated by the active vitamin D metabolite calcitriol [189]. Since CaR transcription is strongly upregulated by vitamin D [190], a Ca^2+^-containing diet could protect against colon cancer partly via upregulation of CaR expression. The work by the group of Enikö Kallay provided clear evidence about the role of dietary Ca^2+^ and dissected the reciprocal interplay between extracellular Ca^2+^/CaR and the Vitamin D system (for an exhaustive review refer to [189]). Significant reduction in CaR expression level has also been observed in unfavorable neuroblastomas when compared with benign, differentiated neuroblastic tumors. However, information about the mechanisms underlying the loss of CaR expression is still scattered and thus under intense investigation. Yet, in colon cancer and neuroblastomas, genetic or epigenetic mechanisms, such as hypermethylation and histone deacetylation of CaR promoter or monosomy of the chromosome 3, have been proposed [9,191].

On the other hand, in prostate and breast tumors, the expression level of the CaR is increased [192,193]. This experimental evidence is in line with an oncogene-like behavior of the CaR (e.g., increasing proliferation and inhibiting cell death), which seems strictly related to the Gαs/cAMP-dependent production of the parathyroid hormone-related protein (PTHrP). Strikingly, in normal mammary epithelial cells, during lactation, the CaR inhibits PTHrP synthesis and secretion through a Gαi, clearly indicating the ability of the receptor to bind and activate opposing G proteins in normal and breast cancer cells [157]. In addition, in breast cancer cells, CaR signalling through Gα12/13 affects cell migration via rho-dependent actin filament formation facilitating the metastatic spread of the tumor [194]. These tumors often lead to bone metastasis, since bone provides homing signals (such as PTHrP-induced high extracellular Ca^2+^) to several cancers, such as breast, prostate, lung, and kidney cancer.

#### 4.5.2. The CaR in the Heart

Another field of emerging interest for its implications on human health concerns CaR function in cardiovascular physiology and pathology.

The CaR has been demonstrated to exert a fundamental role in the vasculature and to affect blood pressure in different experimental models [160,195,196,197,198]. Nice reviews on this topic have been recently published by experts in the field [199,200,201,202].

Hence, the focus of this section is on the specific role of the CaR in the heart, a topic which is still quite controversial and, in our opinion, needs further study in the near future.

The first paper describing the functional expression of the CaR in the heart was published 15 years ago [177]. In that first report, the authors identified CaR mRNA and protein both in atrial and ventricular tissues from adult rats. Also, a tentative imaging approach was performed by using high extracellular Ca^2+^ concentrations, spermine (1–10 mM), and gadolinium on isolated adult ventricular cardiomyocytes. Although the interpretation of data collected with high extracellular Ca^2+^ on Fura-2-loaded cells could be hampered by a protocol in which the cardiomyocytes were first bathed in 0 Ca^2+^, thus activating a serious Ca^2+^ overload upon Ca^2+^ re-addition, the authors provided evidence of effects of extracellular calcium, spermine, and gadolinium on changes of Inositol Phosphate (IP) levels, thus implicating the PLC/InsP3 signalling pathway in CaR activation.

The existence of a functional CaR was next clearly demonstrated in neonatal rat ventricular cardiomyocytes by Tfelt-Hansen and collaborators [178]. Importantly, in this study the calcimimetic AMG 073 was used to activate the CaR and proven to be effective both in IPs accumulation and ERK1/2 activation. Also, the involvement of CaR in IPs modulation was straightforwardly demonstrated by the significant inhibition of Ca^2+^-induced IP accumulation upon infection of neonatal cardiac myocytes with adeno-associated viruses containing the dominant negative CaR R185Q.

Starting from 2006, a conspicuous number of papers has been published by researchers from the Harbin University, where the first paper on the CaR in cardiac tissues was published [177]. The results collected during the last decade by this group depict a general scenario in which CaR activation is mostly causative of cardiac diseases.

On the contrary, other groups have provided evidence of a cardioprotective role of the CaR. Various experimental models and approaches have been used to inquire into the CaR function in cardiac apoptosis induced by different drugs or by ischemia/reperfusion, hypertrophy and fibrosis, and diabetic cardiomyopathy. The role of the CaR in pre- and post-conditioning has also been investigated.

##### Role of the CaR in Cardiac Apoptosis

A pro-apototic effect of GdCl3, used as the only CaR agonist (in the presence of NiCl2 and CdCl2 to block L-type Ca^2+^ channels and Na+/Ca^2+^ exchangers, respectively) was reported by Sun and colleagues in neonatal cardiac myocytes [203]. CaR pro-apoptotic action was suggested to be mediated by intracellular Ca^2+^-induced activation of extracellular signal-regulated protein kinase (ERK), c-Jun NH2-terminal protein kinases (JNK), and p38. GdCl3 was also shown to activate caspase 9 under these experimental conditions.

In parallel, another work was published on isolated rat hearts subjected to an ischemia/reperfusion protocol [204]. By using GdCl3 as CaR agonist, the authors suggested that an increased CaR expression is involved in the induction of cardiomyocytes apoptosis via Ca^2+^ overload-induced cytochrome C/caspase 3 axis activation. Later, Sun and colleagues suggested, by using the same experimental protocol, that one of the molecular players involved in CaR-mediated apoptosis in neonatal cardiomyocytes is the Ca^2+^ channel TRPC6 [205], whose expression was found, like the CaR, to be increased after Gd^3+^ stimulation. Similarly, an increase of TRPC3 expression was described in neonatal rat cardiomyocytes in a following paper, although no evaluation of apoptosis was performed in this study [206].

The effects of known proapoptotic substances were also investigated. Ciclosporin A (CsA) was described to exert its adverse effects (also) by increasing CaR expression both in vitro [207] and in vivo [208].

In neonatal rat cardiomyocytes [207], the proapoptotic role of the CaR in CsA-induced apoptosis was investigated by using Gd^3+^ and NPS-2390 as CaR agonist and inhibitor, respectively. While in the neonatal cell model the CsA-induced CaR-mediated cell death was attributed to Ca^2+^ overload [207], in a similar work performed two years later on H9c2 cardiomyoblasts the involvement of the MAP kinases pathway was suggested [209]. In the same year, the CaR was also implicated in lipopolysaccaride (LPS)-induced apoptosis via its increased expression and consequent Ca^2+^ overload and TNF alpha and IL-6 release [210].

##### Role of the CaR in Apoptotic Pathways Activated by Ischemia/Reperfusion

During the years 2006–2011, some of the same researchers from Harbin University made a considerable effort to disclose the involvement of the CaR in the apoptotic pathway activated by ischemia/reperfusion (I/R) or hypoxia/reoxigenation protocols both on in vivo and in vitro models, i.e., neonatal rat cardiomyocytes [211,212,213,214], isolated adult rat cardiomyocytes [204,215], and adult rat hearts [212,215]. Altogether, the collected results suggest that I/R induces a significant increase of CaR expression. Subsequent CaR-mediated signalling pathways activate apoptosis. The involvement of mitochondria in such an effect was suggested to be mediated by Ca^2+^ overload via inter-organelle signalling [213], ER stress [212], and PKC delta [216]. Activation of the C-Jun NH2 terminal protein kinase signalling pathway was also described as one of the mechanisms relevant for CaR-mediated injury during ischemia/reperfusion in adult rats [215].

Later, another group at the Nanjing University confirmed the work of Jiang et al. [211,217] and implicated a downregulation of CaR expression and signalling in the anti-apoptotic effect of hepatocyte growth factor in neonatal cardiomyocytes subjected to simulated ischemia/reperfusion [218].

##### Role of CaR Inhibition in Post-Conditioning

Almost in the same years, Zhang et al. [219] suggested on isolated rat hearts subjected to an ischemia/reperfusion protocol followed by post-conditioning (PC) that PKCε is involved in the protective effect of the PC maneuver via its negative feedback on the CaR and the subsequent reduction of intracellular Ca^2+^. Also, in these studies GdCl_3_ + NiCl_2_ + CdCl_2_ was the only experimental maneuver used to activate the CaR.

Similar results were obtained some years later on the neonatal rat cardiomyocytes model, this time using also the calcilytic NPS 2390 and implicating the attenuation of CaR-induced sarcoplasmic reticulum–mitochondria crosstalk in the protective effects of PKCε during post-conditioning [220].

In 2012, in a work performed in vivo and on isolated adult rat cardiomyocytes subjected to a hypoxia post-conditioning protocol, the authors suggested that the pro-apoptotic action of CaR exerted via ER stress activation was counteracted during post-conditioning by a reduction of CaR protein expression [221]. Very recently, Zhang and colleagues confirmed that ischemic post-conditioning and KATP channel agonists diazoxide and pinacidil exert a protective effect against ischemia/reperfusion injury through a mitigation of Ca^2+^ overload induced by downregulation of the CaR [222].

Given the relevance of CaR expression levels for the rationale of these studies, it is worth mentioning a recent short communication from the group of Schluter [223] focused on the assessment of CaR expression levels after ischemia reperfusion and cardiac pre- and post-conditioning. This report pointed out that different CaR regulation patterns seem to exist between left and right ventricular tissues and that also the size of infarcts could be a determinant in regulating CaR expression [223].

##### Role of the CaR in Pre-Conditioning

In opposition to the theory of a deleterious action of the CaR in the heart, there was already in 2010 the work by Sun and Murphy in which the authors suggested a cardioprotective role for the CaR, strategically localized in caveolae, during ischemic pre-conditioning (IPC) [224].

In this study, the perfusion of adult rat hearts with the CaR antagonist NPS 2143 during the application of the ischemic pre-conditioning protocol was indeed able to attenuate cardioprotection of IPC, as assessed by measuring the levels of the cardioprotective phospho ERK1/2, AKT, and GSK3β kinases, infarct size, and functional recovery. The suggestive speculation of the authors depicts a scenario in which the changes in pH and ionic strength imaginable at the particular microdomains of caveolae may activate the CaR, which could thus “sense” and “mediate” IPC.

Another controversial topic is the role of CaR activation in the progress of diabetic cardiomyopathy [225,226].

On the one hand, Bai et al. suggested that CaR expression is reduced in diabetic rat hearts and could contribute to the progress of diabetic cardiomyopathy via impaired intracellular Ca^2+^ signalling in adult cardiac cells [225]. On the other hand, Qi and colleagues showed that CaR expression is rather increased in diabetic rat hearts and by using Gd^3+^, NPS 2390, and a siRNA to silence CaR protein, suggested that CaR signalling contributes to apoptosis of neonatal cardiomyocytes exposed to high concentrations of glucose in vitro via Ca^2+^ overload, reduction of the Bcl2/Bax ratio protein expression, and modulation of the MAP kinase pathway [226]. The apparent discrepancy of results on CaR expression was explained by invoking a difference in the experimental models which, according to Qi et al., were representative of type 2 diabetes in the first work [225] and type 1 diabetes in the latter [226].

##### Role of the CaR in Hypertrophy and Heart Failure

A role for the CaR in cardiac hypertrophy has been postulated since 2006, when Tfelt-Hansen and collaborators [178] observed a CaR-induced decrease in DNA synthesis in neonatal cardiomyocytes. Considering that this parameter increases in cells undergoing hypertrophy [227], the authors supposed a protective role for the CaR against cardiac hypertrophy.

In opposition to this theory, in a study performed by Wang and colleagues [228] on an in vitro model of hypertrophy, an increased expression of the CaR was found and exacerbating effects of Gd^3+^ on Ca^2+^ signalling and hypertrophic markers were argued to be mediated by the CaR.

Later, Zhong et al. suggested a role for the CaR in the Gd^3+^-induced increase of nuclear Ca^2+^ spikes’ frequency and consequent activation of the Calcineurin/NFAT pathway [229], a well-known player in cardiac hypertrophy [230].

The role of the CaR in cardiac hypertrophy and heart failure was further investigated in a successive work [231], in which Calindol and Calhex 231, as agonist and inhibitor of the CaR, respectively, were subcutaneously injected in isoproterenol-treated Wistar rats to assess the role of the CaR in ER stress in vivo. CaR expression was found to significantly increase during heart failure induced by isoproterenol and Chalex 231 significantly reduced the cross-sectional diameter of hypertrophic cardiomyocytes. In the same study, the CaR was suggested to play a role in cardiac hypertrophy and heart failure also in mice subjected to thoracic aorta constriction (TAC) [231]. In this model, Calindol was found to induce significant cardiac hypertrophy.

The involvement of the CaR in ER stress-mediated apoptosis in failing hearts was also suggested by the observation that Calindol and Chalex 231 were able to increase and decrease, respectively, ER stress protein expression and apoptosis in both the rat and mouse models. Finally, a CaR-induced IP3-dependent Ca+ overload of mitochondria and subsequent initiation of apoptosis were suggested in rat hearts and neonatal rat cardiomyocytes incubated for 48 h with isoproterenol. On these bases, the authors proposed that the CaR could represent a putative target for pharmacological intervention in heart failure.

Very recently, Liu et al. suggested the possibility to ameliorate isoproterenol-induced cardiac hypertrophy through suppression of CaR signalling with the CaR inhibitor Chalex 231 both in vivo and in vitro [232]. In this paper, the authors suggested that the anti-hypertrophic effect and amelioration of cardiac function by Chalex 231 may be mediated through inhibition of autophagy and the CaMKK-AMPK-mTOR signalling pathway.

A recent in vitro and in vivo study also suggested a role for the CaR in promoting cardiac fibrosis [233].

In opposition to a maladaptive pro-hypertrophic effect of the CaR, which is the common denominator of the above-described studies, there are recent contributions from the group of Schluter [223] which sustain the theory of a cardioprotective effect of the CaR in a pharmacological model of hypertension in adult rats. Interestingly, these authors showed that upon NO-synthase inhibition an increased expression of endothelin A and/or B1 receptors and CaR was measurable. This upregulation of CaR, most probably mediated by a paracrine action of endothelin 1, was suggestive of a CaR-induced adaptive hypertrophy. Indeed, CaR inhibition or destabilization by NPS 2390 and siRNA against RAMP, respectively, decreased load-free cell shortening, which was used as a read-out of cardiac function.

##### Role of the CaR on the Electrical Properties of Cardiac Cells

Notwithstanding the abrupt proliferation of literature on the role of the CaR in the heart in the last 15 years, very few data were available, until recently, on the effect of CaR agonists and modulators on the electrical properties of cardiac cells.

In very recent times, the group of Schluter [234] and Shieh [235] have published interesting data on this topic on adult rat hearts and guinea pig cardiomyocytes, respectively, by using different physiologically relevant experimental approaches.

First, Schreckenberg and colleagues examined the effects of CaR acute stimulation on the cardiac physiology of adult male rats [234]. The experimental approaches were diverse: the contractile response of ventricular cardiomyocytes was recorded with a cell-edge-detection system in parallel with Fura-2 measurements of cytosolic Ca^2+^, while cardiac performance in response to acute CaR stimulation or inhibition was assessed on left ventricle muscle stripes and whole hearts by the Langendorff system.

Putrescine and Gd^3+^ were used as CaR agonists, while NPS-2390 (10/100 µM) and downregulation of CaR by siRNA were used to assess the specificity of the responses.

The data collected, while confirming the expression of CaR by adult rat ventricular cardiomyocytes, demonstrated that CaR action is relevant for basal cell shortening at physiological extracellular Ca^2+^ concentrations and that additional activation of the CaR speeds up cardiomyocytes relaxation and augments cell shortening, supposedly via increased Ca^2+^ transients induced by activation of the Gq/PLC/IP3 pathway.

Very interestingly, Liu et al. clearly demonstrated that activation of the CaR in guinea pig cardiomyocytes by the allosteric agonist NPS R 568 activates both the PLC and phsphatidylinositol-4 kinase (PI4) Kinase pathways and that the PI4 Kinase pathway predominates [235]. The net effect is an increase in PIP2 at the plasma membrane, which in turn determines a significant increase in currents through inward rectifier K+ channels. This action could thus exert a stabilization of the resting membrane potential of cardiomyocytes and contribute to a re-evaluation of old data on the stabilizing and cardioprotective effects of polyamines [236].

Finally, interesting hints on the CaR’s role in the heart come from a paper focused on the function of the CaR in regulating blood vessel tone and blood pressure [237].

Since the experimental model of CaR KO mice (SM22αCaSRΔflox/ΔfloxKO mice) used in this work also display a deletion of the CaR in cardiac cells, the authors tested the hearts of KO mice by perfusion in the Langendorff mode. Given the significantly reduced basal heart rate found in KO mice versus wild-type (WT) animals, the authors suggested that CaR deletion from heart directly affects chronotropy. Also, ex vivo lung slice preparations from KO mice showed a reduced rate of spontaneous activity, suggesting that the decreased chronotropy in KO mice could be due to decreased pacemaker activity.

In summary, notwithstanding the numerous results collected on physio-pathological roles of the CaR in the heart, the current findings are far from providing a coherent picture.

Considering CaR promiscuity and pleiotropicity and the emerging concept of biased signalling at the CaR (see below), an interesting field of future investigation would be the accurate pharmacological assessment of the effects exerted by panels of CaR agonists and modulators on different signalling pathways in physiologically relevant experimental models.

Given the actual and potential use of calcimimetics and calcilytics in the clinic, these studies would be highly significant for their implication for human health.

### 4.6. Novel Trafficking/Signaling Modes of the CaR

Although GPCR have often been seen as cell surface proteins able to go through a simple on-off switch, research of the past few years has drawn a much more complex scenario, in which receptors exist in multiple conformations and, in dependence of the different ligands, can be stabilized in different conformational states, in turn activating multiple, yet specifically selected, downstream pathways whose identity depends on the combination of ligands and the specific signalling toolkit of the cell where they are expressed [238,239,240,241,242].

The development of new technologies, such as microscopy techniques and probes to follow receptor trafficking and subcellular signal dynamics (such as FRET and single molecule microscopy) as well as BRET-based high throughput assays for biased signalling [243], have been essential to demonstrate that these proteins are capable of trafficking among a variety of subcellular compartments, initiating specific signalling pathways at different locations: nuclear [244], mitochondrial [245], and endosomal–Golgi membrane [246,247].

As stated in the paragraph regarding the structural features of the CaR, functional receptors localized at the plasma membrane are dimers of the glycosilated CaR which are formed intracellularly [248] and are stabilized by ligand binding at the cell surface [249]. As for other GPCRs, the expression level of CaR is the result of a fine regulation of the dynamic equilibrium between mechanisms that precede its biosynthesis (transcription/mRNA level, translation efficiency, epigenetic control [250,251]) and regulate its anterograde trafficking and those controlling its retrograde trafficking, including degradation. In fact, it has been known since a long time that the fine balance between maturation, internalization, recycling, and degradation can influence the net level of cell surface receptor and thus represent a mechanism for the cell to regulate receptor desensitization and modulate strength of signal transduction [252]. Thus, the strength of signaling depends upon the level of GPCRs expression at the plasma membrane and the consequent receptor availability for agonists and modulators. This is also the case for CaR signalling as recently established by Conigrave’s group [253].

A significant contribution to the field of CaR trafficking has come in the last ten years from different groups who have focused on the elucidation of the key steps involved in CaR biosynthesis and trafficking from the ER membrane to the Golgi membrane and then to the plasma membrane (see [254] for a recent review). A fundamental role in this field is also played by research focused on the CaR-interacting proteins, which finely modulate anterograde and retrograde trafficking of the CaR (for comprehensive reviews see [10,133,254,255,256]). 

#### The Agonist-Driven Insertional Signalling (ADIS)

Both early and recent studies have revealed two peculiar characteristics of the CaR: a minimal functional desensitization (both by phosphorylation and/or β-arrestins) and a significant “reservoir” of CaR proteins in intracellular membrane compartments. Western blotting or immunohistochemistry analyses [11,76,257] have clearly showed, since early studies, that CaR immunoreactivity is predominantly represented by an intracellular, core-glycosilated form. Recent data are making increasingly clear that such an observation is not an artifact but is strictly related to the complex interaction between CaR trafficking and signalling. In fact, both the hallmarks of the CaR, minimal desensitization and high levels of intracellular proteins, can be explained by a novel signalling mode described by the group of Dr. Breitwieser and termed Agonist-driven insertional signalling (ADIS) [250,258].

In this model, CaR signalling is regulated by an agonist-dependent modulation of the net amount of receptor proteins at the plasma membrane. In particular, the level of CaR cell-surface expression results from the balance between the rate of trafficking from a large, pre-plasma membrane pool located at the Golgi/trans-Golgi/post-Golgi vesicles (which depends upon the concentration of CaR agonists and/or allosteric modulators) and the process of constitutive endocytosis of the receptor already at the plasma membrane. The sensitivity of parathyroid and renal tubular cells to extracellular Ca^2+^, for example, is likely influenced by the balance between ADIS, which enhances anterograde trafficking of newly synthesized CaR proteins to the plasma membrane [258] and clathrin-mediated endocytosis and retrograde trafficking of cell-surface CaR receptors [10].

### 4.7. Synthetic Allosteric Modulators of the CaR Can Function as Pharmacoperones

Strictly related to the research in the field of CaR trafficking is the observation that CaR allosteric modulators (see Paragraph 4.3) can work as pharmacoperones. Pharmacoperones are permeant substances (which could be agonists, antagonists, or allosteric modulators) that can reach a misfolded protein at its intracellular location (most commonly the endoplasmic reticulum), stabilize it, and rescue the receptor to the cell membrane surface.

A relevant effort on the capacity of CaR allosteric modulators to work as pharmacoperones comes from Breitwieser’s group [259,260,261]. These authors firstly demonstrated that the positive allosteric modulator NPS-R-568 was able to rescue almost fifty percent of the loss-of-function mutant CaRs analyzed [259,260]. In parallel, another group demonstrated the same ability of NPS-R-568 to rescue intracellular signalling in four mutants (out of seven) although the plasma membrane localization of the receptors was not assessed [262].

In the last five years, relevant findings in the field of pharmacoperone action of type II calcimimetics and calcilytics have been provided by studies from the group of Dr. Leach. These authors, while confirming data on NPS-R-568, showed the ability of cinacalcet, the only CaR modulator approved in the clinic, to effectively rescue trafficking and signalling of CaR mutants with loss of cell surface expression. [128,263]. They also showed that NPS-2143, a negative allosteric modulator, is able to positively modulate CaR trafficking to the cell membrane while negatively modulating CaR signalling in the presence of the orthosteric substrate [144,263], underlining a striking example of positive bias (see also above) toward trafficking by a CaR allosteric modulator.

Nonetheless, both early and recent studies suggest that the pharmacoperone action of NPS R-568 [264] and NPS-2143 [259,264] is mutant-specific.

Importantly, the effectiveness of old and new calcilytics on gain-of-function mutations of the CaR was further explored in vitro [265,266], demonstrating that a novel treatment option for patients with activating CaR mutations could be represented by the new quinazolinone-derived calcilytics, which have been shown to be effective in attenuating enhanced cytosolic Ca^2+^ signalling in four known mutations causing Bartter syndrome (BS) type 5 and autosomal dominant hypocalcemia (ADH). Very interestingly, recent in vivo works involving a mouse model harboring an ADH gain-of-function CaR mutation demonstrated the effectiveness of both old (i.e., NPS 2143) [267] and new (i.e., JTT-305/MK-5442) [268] calcilytics in normalizing the defect and improving the associated hypocalcemia.

### 4.8. Biased Signalling

Biased signalling (also termed biased agonism or ligand-directed signalling or stimulus bias) represents a recently described, yet quite general, modality of the signalling characteristic of diverse GPCRs [240].

It concerns the capability of different ligands to stabilize diverse conformations of the receptor and thus preferentially head GPCR signalling towards a specific transduction cascade.

This new concept, which substitutes the idea of one receptor/one GPCR conformation/one-uniform activation of signaling pathway(s), greatly complicates the scenario of GPCRs signalling, but opens new perspectives in the design of smart and tissue-specific drugs that avoid off-target actions or on-target effects on multiple tissues [269].

Given the promiscuity and pleiotropicity of the CaR and the wide expression of this receptor in tissues involved or not in the regulation of systemic Ca^2+^, it is not surprising that fundamental insights into this new ligand- and tissue-specific pharmacology come from the study of biased signalling at the CaR.

In fact, the existence of ligand- and tissue-specific effects in the signalling pathways activated by the CaR, although not precisely quantified/measured, are traceable in a vast number of papers published throughout the years.

It could also be hypothesized that the experimental approaches used in the recent past could have contributed to an underestimation of the phenomenon of biased signalling at the CaR. Most of the work on CaR signalling is in fact performed by testing only a small number of CaR agonists and modulators on a limited array of signal transduction cascades (most frequently cytosolic Ca^2+^ dynamics). A striking example of biased signalling at the CaR comes from an acquired hypocalciuric hypocalcemia autoantibody, which was shown to increase the effects of external Ca^2+^ on Gq signalling to the detriment of the Gi-induced signalling pathway [270]. As far as the differential effects of CaR agonists and modulators are concerned, both early studies by Riccardi’s group on rat pancreas [271] and more recent work by Ziegelstein and colleagues on human aortic endothelial cells showed that only some CaR ligands were able to induce intracellular Ca^2+^ release, while others were ineffective [160]. Also, at the level of physiological functions, Tfelt-Hansen’s group demonstrated that while cinacalcet was able to induce vasodilatation of rat aorta, the neomycin and Gd^3+^ were ineffective [161]. 

In the last years, a large number of findings has highlighted the physiopathological significance of biased signalling.

A major contribution in this area has come from Bräuner-Osborne’s group [145,272,273]. Specifically, these authors explored the action of a panel of well-known orthosteric CaR agonists on the Gq/11 protein, Gi/o protein, and extracellular signal-regulated kinases 1 and 2 (ERK1/2) pathways. Thomsen and colleagues [145] revealed that in HEK-293 cells stably transfected with rat CaR, the natural agonist Ca^2+^ is biased toward cAMP inhibition and IP3 accumulation over ERK1/2 phosphorylation. Differently, a significant bias toward ERK1/2 phosphorylation was shown for the polyamine spermine.

Interestingly, while two different groups showed that strontium ranelate, a drug used as treatment for osteoporosis, is a biased agonist of the CaR toward the ERK1/2 pathway and another cascade independent of Gq/11 signalling [273,274], different results were reported by others [275]. In another study, while both Sr^2+^ and Ca^2+^ were shown to stimulate PLC and NF-kB, only strontium ranelate induced apoptosis via PKC activation [276].

In recent years, the possibility of exploiting and “inducing” stimulus bias at the CaR has been deeply explored also by the group of Christopoulos and Leach [95,263,277,278] (reviewed in [144,269]). The first quantitative evidence that an allosteric modulator used in clinical practice exhibits stimulus bias was provided a few years ago by Davey and colleagues [277], which straightforwardly proved that allosteric modulators are biased toward intracellular Ca^2+^ signalling in a HEK293-TREx c-myc-CaR cell line.

Next, the same group analyzed in detail the effect of CaR allosteric modulators on naturally occurring CaR mutations [278], demonstrating that mutations can alter signalling bias by stabilizing receptor conformations that differentially couple to downstream signalling pathways. Importantly, and as stated above, in a subsequent paper, both Cinacalcet and the calcylitic NPS-2143 were demonstrated to have a bias toward Ca^2+^ signalling and rescue CaR mutants to the cell membrane [263]. The therapeutic implications of such data are enormous.

Just as an example, the calcimimetic cinacalcet has been used in clinic for the treatment of patients carrying loss-of-function CaR mutations only in the cases of end-stage renal disease. This is because of its hypocalcemic properties, most probably due to the Ca^2+^-mediated increase of calcitonin secretion [272]. It is thus clear that designing a novel drug able to act on parathyroid glands without affecting calcitonin secretion from parafollicular cells would be the best therapeutic option.

Remarkably, in a very recent paper, the search for a calcimimetic able to suppress PTH levels at concentrations with low/no effect on serum calcitonin seems to have found good candidates among the third generation of calcimimetics [128]. Altogether, these findings highlight the importance of a deeper comprehension of the mechanisms of action of different orthosteric agonists and modulators in the different tissues for the better design of drugs that are devoid of side effects and are able to activate specific pathways in a cell-selective fashion [144,269].

It is worthwhile noting that an even higher level of complexity in the scenario of biased agonism and pharmacoperones action of CaR ligands is the observation that GPCRs have been recently shown to sustain G (Gs or Gi) protein signalling at different subcellular sites even after internalization of ligand–receptor complexes in endosomes (for a recent review see [242]).

## 5. Old and New Extracellular Ca^2+^ Sensors

Even though the CaR is so far the best-studied extracellular Ca^2+^ sensor, other receptors (either membrane proteins or channels) able to change their function following significant fluctuations in extracellular Ca^2+^ level have been identified [279]. Of note, those of the same family of the CaR have been the focus of intense studies based on a high degree of structural homologies, conservation of specific domains, and a common functional role as nutrient/salinity sensor. This family includes eight metabotropic glutamate receptors (mGluR1–8), two gamma-aminobutyric acid (GABA) receptors, three taste receptors (T1-T3), the recently “deorphanized” GPRC6A, and seven orphan receptors [280].

### 5.1. Metabotropic Glutamate Receptors

Metabotropic glutamate receptors (mGluRs) are neurotransmitters expressed mainly in the central nervous system, where they modulate key functions in a variety of different neurological processes (e.g., memory, learning, pain, and synaptic plasticity) [281]. mGluRs (especially mGluR1 and -R5) possess significant sensitivity to extracellular Ca^2+^ [282,283]; however, it is still unclear whether they exist in nature as proper Ca^2+^ sensors [282] or whether extracellular Ca^2+^ just sensitizes the receptor to its principal ligand, the excitatory amino acid l-glutamate [284,285]. For example, Ca^2+^ depletion in the synaptic cleft during burst firing induces significant inhibition of postsynaptic mGluR1 function [286]. In addition, Gerber et al. showed that increasing concentrations of extracellular Ca^2+^ might enhance the potency/efficacy of glutamate action on mGluRs1 [287]. Recently, a novel potential extracellular Ca^2+^-binding site was identified within the extracellular domain of the mGluR1 adjacent to the known l-Glu-binding site [288,289]. When both l-Glu and Ca^2+^ are bound to their partially overlapping binding sites, they synergistically modulate mGluR1 activation. Strikingly, a significant reduction in the sensitivity of mGluR1 to extracellular Ca^2+^ and, in some cases, to l-Glu is induced by a mutation in the predicted Ca^2+^-binding residue [290]. Furthermore, this Ca^2+^-binding site is also adjacent to the interaction sites of orthosteric agonists and antagonists; thus, extracellular Ca^2+^ might modulate the sensitivity of mGluR1 not only to orthosteric agonists and antagonists but also to allosteric modulators [291].

### 5.2. The GABA_B_ Receptor

The gamma-aminobutyric acid subtype B (GABA_B_) receptor plays a key role in regulating membrane excitability and synaptic transmission in the brain. The functional receptor consists of a heterodimer of GABA_B-R1_ and GABA_B-R2_ [292]. As for the CaR and mGluRs, the GABA_B_ receptor has the ability to sense extracellular Ca^2+^ (0.001–1 mM) but not other divalent and trivalent cations. Wise et al. showed that an addition of 1 mM extracellular Ca^2+^ potentiated GABA stimulation (EC50 was reduced from 72 to 7.7 μM) in membrane preparations from both CHO cells (that stably express the GABA_B-R1/R2_ heterodimer) and rat brain cortex. Of note, even though Ca^2+^ does not act as a direct agonist of the receptor, extracellular Ca^2+^ may allosterically enhance the GABA-induced responses of the GABA_B_ receptor [293].

### 5.3. Taste Receptors

T1R1 and T1R3 belong to the T1R family and form an heterodimer that is considered a broadly tuned l-amino acid sensor. Silve et al. reported a Ca^2+^-binding pocket for T1R3. Of note, this receptor subunit is characterized by Ca^2+^-binding residues that are conserved in the Venus fly trap region of the CaR [294]. Recently, in vivo taste studies have suggested the involvement of T1R3 in Ca^2+^-Mg^2+^ taste in mice and humans [295,296]. These studies have also supposed that T1R3 might form a dimer with another receptor able to sense extracellular Ca^2+^, such as the CaR.

### 5.4. The G Protein-Coupled Receptor Family C Group 6 Member A (GPRC6A)

The G protein-coupled receptor family C group 6 member A (GPRC6A) displays the closest mammalian homology to the CaR. This receptor, cloned in 2004, is extensively expressed at the tissue level [297] while limited information is available at the sub-tissue level. So far, experiments with KO mice for the receptor have shown GPCR6 involvement in the regulation of biological processes such as inflammation, metabolism, and hormonal functions. GPCR6 is activated by a large number of basic and small aliphatic l-α-amino acids (being l-arginine, l-lysine, and l-ornithine the most potent compounds). In addition, high extracellular Ca^2+^ levels (5–10 mM) either directly activate or positively modulate the receptor. This range of extracellular Ca^2+^ concentration can be physiological only in a tissue, such as the bone, where the receptor seems to be involved in bone remodelling processes [298]. However, it remains unclear which of the proposed agonists are the most important physiologically. Furthermore, CaR allosteric modulators, such as NPS568, Calindol, and NPS2143 (IC50~10 μM), were shown to be effective also on GPRC6A [67,298,299].

### 5.5. Notch Receptor

The epidermal growth factor (EGF)-like domain contains a Ca^2+^-binding domain with a wide range of Ca^2+^ affinities [300]. This type of module is probably the most prevalent extracellular Ca^2+^-binding site. Among the proteins containing Ca^2+^-binding EGF-like domains, there are proteins involved in blood coagulation, fibrinolysis and its complement system, matrix proteins, and the developmentally important cell surface receptors Notch [300]. Left–right asymmetry in all vertebrates is established during embryo development by several different molecular events. Raya et al., using a combination of modelling and experimental procedures, showed that, in chick embryos, asymmetric patterns of gene expression were the result of an extracellular Ca^2+^ gradient toward the left margin of the developing embryo. Here, extracellular Ca^2+^-induced activation of Notch results in the asymmetric secretion of Nodal, a specific signalling molecule [301]. Of note is that the perturbed establishment of the left–right patterning in embryo maturation was induced by the disruption of the extracellular Ca^2+^ gradient.

### 5.6. Cadherins

Cadherins are a family of Ca^2+^-dependent adherent receptors involved in development, morphogenesis, synaptogenesis, differentiation, and carcinogenesis. Most of the members are single membrane-spanning proteins possessing Ca^2+^-binding extracellular domains with a wide range of Ca^2+^ affinities (Kd from μM to mM) [302]. The cadherin extracellular domains act as a Ca^2+^-activated “mechanical switch” through which extracellular Ca^2+^ fluctuations are detected and integrated to control their mechanical integrity and mechano-transduction capability.

### 5.7. Stromal Interaction Molecule 1 (STIM1)

Ca^2+^-binding proteins containing EF-hand domains are present in the extracellular environment or matrix. These proteins do also localize at the plasma membrane, with the EF-hand domain facing the extracellular medium [303,304]. An important example of this class of proteins is the single transmembrane-spanning Ca^2+^-binding protein, STIM1, generally known as the endoplasmic reticulum Ca^2+^ sensor, which provides the trigger for store-operated channels (SOCs) activation. STIM1 at the cell surface may represent a fundamental pharmacological target to finely tune Ca^2+^-regulated functions that are mediated by SOCs.

### 5.8. Hemichannels

Gap junctions are constituted by the juxtaposition of two membrane-spanning connexons (hemichannels) positioned end-to-end. However, functionally active hemichannels exist at the plasma membrane where they allow the rapid bi-directional movement of ions, second messengers, and metabolites between the cytosol and the extracellular space and vice-versa [305]. Opening of most hemichannels depends critically on decreases in extracellular Ca^2+^ levels (of about ~200 μM) around the physiological Ca^2+^ levels [306,307]. The vestibule of Connexin 32 has a Ca^2+^-binding site involved in the regulatory effect of external Ca^2+^. Of note is that it has been shown that the complete Ca^2+^ deregulation of connexin 32 hemichannel activity is induced by a mutation causing an inherited peripheral neuropathy. This evidence led to the assumption that the pathogenesis of the neuropathy may be strictly related to this dysfunction [308]. The pore of connexin 43 hemichannels might be in an open or closed conformation, with the open probability significantly dependent on external Ca^2+^ levels (from 1.8 to 1.0 mM) [309].

### 5.9. Ion Channels

Dynamic changes in extracellular Ca^2+^ are known to impact on a number of ion channels, especially those located in the brain.

Ca^2+^ homeostasis modulator 1 (CALHM1) is an ion channel expressed at the cell membrane of cortical neurons and type II taste bud cells of the tongue [310]. This peculiar channel, closed at resting membrane potential and modulated by significant membrane depolarizations, is also able to sense a decrease in extracellular Ca^2+^ that thus works, in synergy with voltage, to allosterically increase the CALHM channel gating open probability.

Interestingly, Ca^2+^ and other cations, anions, and ATP are able to permeate through the non-specific pore of CALHM1 [310].

Acid-sensing ion channels (ASICs) are trimeric cationic channels activated by extracellular protons and primarily expressed in the nervous system. These channels are permeable to Na+ and Ca^2+^ and “sense” extracellular Ca^2+^, which modulates their open probability by competing with protons [311,312,313]. In addition, two negatively charged residues near the entrance of the channel pore have been shown to be critical for the direct Ca^2+^ blockade of ASIC1a currents [313]. Of note is that these channels seem to be also modulated by small fluctuations in extracellular Zn^2+^, Mg^2+^, and spermine [311,314].

Another class of ion channels modulated by dynamic changes of extracellular [Ca^2+^] are the voltage-gated Human ether-à-go-go-related gene (HERG) encoded K+ channels, which have been shown to be implicated in a variety of physiopathological mechanisms, such as neuronal spike frequency adaptation and tumor cell growth [315]. Johnson et al. showed that small changes in extracellular Ca^2+^ under physiological conditions shift the voltage dependence of channel activation to more positive membrane potentials, allowing for fine-tuned cell repolarization [316]. Under this scenario, HERG channels seem to be responsible for a delayed rectifier K+ current functional to cellular repolarization of the cardiac contractile cycle in the heart [317].

Finally, the Transient receptor potential cation channel, subfamily M, member 7 (TRPM7) was shown to sense a reduction of extracellular Ca^2+^ in hippocampal and pyramidal neurons and aid an inward cation current [318,319,320]. These 36-pS channels (originally named Ca^2+^-sensing nonselective cation channels, IcsNSC), which are almost silent at the 2.0 mM extracellular Ca^2+^ level, become robustly activated by small decreases in extracellular Ca^2+^ [321].

The history of research on the CaR’s role in physiology and pathological states is a perfect example of the relevance of basic research for ameliorating human health. It is in fact clear that none of the clinically relevant discoveries in the pharmacological field today could have ever taken place without a deep knowledge of the specific mechanisms underlying the functionality of the tissues and organs on whom CaR mutations have their more striking effects.

It is also clear that the novel methodological tools available to researchers in the field of GPCRs and signal transduction will be of paramount importance to further improve the knowledge of basic signalling modes, a step that is indispensable for the design of smart drugs able to rescue GPCR defects in human diseases.

## Figures and Tables

**Table 1:** Orthosteric agonists and allosteric modulators of the calcium-sensing receptor (modified from [110]).

**Orthosteric Agonists (Type I Calcimimetics)**	**Class Members**
A. Cations	High potency: Gd^3+^; Eu^3+^; Tb^3+^
	Intermediate potency: Zn^2+^; Ni^2+^; Cd^2+^; Pb^2+^; Co^2+^; Fe^2+^
	Low potency: Ca^2+^; Mg^2+^; Ba^2+^; Sr^2+^; Mn^2+^
B. Polyamines	spermine, spermidine, putrescine
C. Aminoglycoside antibiotics	neomycin, gentamycin, tobramycin, poromomycin, kanamycin, ribostamycin
D. Basic polypeptides	Poly-L-arginine, poly-L-lysine, protamine, amyloid β-peptides
**Allosteric Modulators (Type II Calcimimetics)**	
A. L-amino acids	phenylalanine, tryptophan, tyrosine, histidine
B. Glutathione analogs	γ-glutamyl-tripeptides: glutathione, *S*-methylglutathione, *S*-propylglutathione, γ-glutamyl-tripeptides (γ-Glu-Ala, γ-Glu-Cys)
C. Small molecule calcimimetics	The first generation: NPS R-568, NPS R-467, AMG 073, AMG 416
	The second generation: cinacalcet
	The third generation: dibenzylamine calcimimetics, *R*,*R*-calcimimetic B, AC-265347
D. Small molecule calcilytics	NPS 2143, Calhex 231, ATF396, AXT914, ronacaleret, NPSP795, SB-423557, SB-423562

## Abbreviations

AA	arachidonic acid
AC	adenylate cyclase
ATP	adenosine triphosphate
CaM	Calmodulin
CaMK	Ca^2+^/calmodulin-dependent protein kinase
cAMP	cyclic AMP
DAG	Diacylglycerol
eNOS	endothelial nitric oxide synthase
ER	endoplasmic reticulum
ERK 1/2	extracellullar-signal regulated kinase
iNOS	inducible nitric oxide synthase
InsP3	inositol-1,4,5-trisphosphate
JNK	Jun amino-terminal kinase
MAPK	mitogen-activated protein kinase
MEK	MAPK kinase
NO	nitric oxide
p38	p38 mitogen-activated protein kinase
PA	phosphatidic acid
PHP	Pharmacoperones
PI3K	phosphatidylinositol 3-kinase
PI4K	phosphatidylinositol 4-kinase
PIP2	phosphatidylinositol 4,5-bisphosphate
PKC	protein kinase C
PLA2	phospholipase A2
PLC	phospholipase C
PLD	phospholipase D
RhoA	Ras homolog gene family, member A
SOC	store-operated Ca^2+^ channel
